# I drank too much and now I can’t walk: a case of alcohol-induced dysautonomia

**DOI:** 10.1093/omcr/omab072

**Published:** 2021-08-13

**Authors:** Richard Assaker, Georges El Hasbani, Arturo Alvarez Antezana, Jose Vargas Gamarra, Jose Amaya-Suarez, Christopher Bertely, Natasha Suleman

**Affiliations:** 1 St. Vincent’s Medical Center, Bridgeport, CT, USA; 2 American University of Beirut Medical Center, Beirut, Lebanon; 3 Lincoln Medical and Mental Health Center, Bronx, NY, USA

**Keywords:** dysautonomia, neuropathy, alcohol

## Abstract

Dysautonomia is a dysfunction of the autonomic nervous system, which mediates both sympathetic and parasympathetic functions of the human body. Alcohol has been established to affect the autonomic function through liver injury and accumulation of vasodilators. Alcohol can induce peripheral neurological diseases as well. This case report describes a patient who had a chronic history of alcoholism and uncontrolled diabetes mellitus presenting for orthostatic hypotension and peripheral neuropathy without underlying liver disease or other endocrinopathies. Although diabetes mellitus was controlled pharmacologically and risk factors for orthostatic hypotension were managed conservatively, his symptoms did not improve which indicated an alcohol-related autonomic dysfunction, shedding light on one of long-term complications of alcoholism.

## INTRODUCTION

The autonomic nervous system, a division of the peripheral nervous system, coordinates the function of internal organs. It is made up of the sympathetic and parasympathetic nervous systems. They are largely responsible for the involuntary, subconscious controls of viscera, smooth muscle and secretory glands [1]. Autonomic dysfunction or dysautonomia is a neuropathy affecting the autonomic nervous system. The effect of alcoholism on autonomic function tests have been well established through the mechanism of liver injury. In this case report, we describe a 36-year-old patient, who struggled with alcoholism, and presented with multiple episodes of orthostatic hypotension found to be a sign of autonomic dysfunction exacerbated by alcoholism. Despite treatment and control of the risk factors, the autonomic dysfunction persisted upon a long period of follow-up.

## CASE PRESENTATION

A 36-year-old male presented to the emergency room with a 3-week history of multiple episodes of dizziness, lightheadedness, blurry vision and tinnitus after standing up. He also reported an episode of near-syncope with trauma to the head 1 day prior to presentation.

The aforementioned presentation was his third in a period of 3 weeks. He endorsed a 40 kg unintentional weight loss in the past 2 years. He had increased sleep, muscle and bone pains, tingling and numbness of his fingers and in his lower extremities distally to his knees.

Past medical history was significant for type 2 diabetes mellitus diagnosed in January 2017 with a hemoglobin A1c (HbA1c) of 7.9% which increased to 10.5% in December 2018 few months before presentation. His diabetes mellitus was being treated with Metformin which he was not compliant with. He also had proliferative diabetic retinopathy, bilateral diabetic peripheral neuropathy treated with Gabapentin, right cerebellar gliosis 2/2 to right frontal ventriculostomy along with a history of depression and alcoholism (reports drinking 10 beers per day for 2 years. Each beer is 24 oz. He quit 3 weeks prior to presentation). He did not report any intake of antidepressant therapy or sedatives.

He is a thin, pale man of Asian descent. There were no signs of dehydration such as dry mouth, lips and eyes. Orthostatic blood pressures: 148/97 mm Hg while sitting and 93/64 mm Hg upon standing. Generally normal body and neurological exams. Neurologic exam was normal. Tilt table test was not performed because the hospital does not have the specific facility for the test. Pertinent initial laboratory findings were: HbA1c 10.2%, AST 87 mg/dL and normal ALT. An EKG showed long QT segment with QTc = 0.53 s.

He was admitted for further work-up of dizziness. Twenty-four hour telemetry were unremarkable. No cardiac echocardiography was performed. No dehydration was suspected due to normal laboratory work-up.

During hospitalization, he was evaluated by an endocrinologist and extensive laboratory studies including PTH, 25-OH vitamin D, vitamin B12, Free T4, ACTH, Cortisol, Testosterone, LH, FSH, Prolactin and IGF-1 levels all returned normal. Serum aldosterone and renin levels were normal excluding adrenal insufficiency as a cause for orthostatic hypotension.

**Figure 1:**
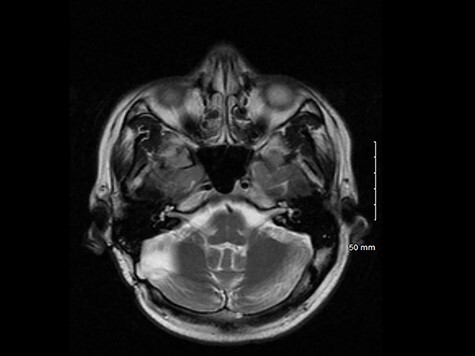
T2 weighted MRI axial image showing right cerebellar gliosis related to a previous surgery.

A computed tomography (CT) scan of the abdomen and pelvis with IV contrast to rule out any liver injury secondary to alcohol use was normal. A CT brain without contrast showed no acute intracranial injury. A right cerebellar gliosis was evident along with prior right frontal ventriculostomy. A brain magnetic resonance imaging (MRI) showed mild gliosis in the lateral aspect of the right cerebellar hemisphere underlying a right occipital craniectomy and a mild right frontal gliosis along a prior shunt tract (Fig. 1).

He worked regularly with physical therapy and was taught posture stabilizing exercises, which improved his functional capacity and ambulatory abilities. He was diagnosed with dysautonomia secondary to alcoholism. His blood pressure improved to 130–140/70–80 mmHg with no significant drop on lying/standing measurements. He was discharged on midodrine 10 mg thrice daily, gabapentin 300 mg thrice daily and thiamine 100 mg daily.

In a 6-month follow-up visit, his HbA1c decreased to 6.5% being on insulin therapy. He denied any further alcohol intake. Although he was compliant to his medications, and was following instructions for management of his varicose veins, he still had intermittent episodes of orthostatic hypotension with blood pressure dropping occasionally to 100/50–60 mmHg. However, he did not report any further near-syncope episode. A gastrointestinal evaluation was scheduled because of significant weight loss.

## DISCUSSION

Alcohol and its metabolites can increase the concentration of vasodilators such as nitric-oxide activating the renin–angiotensin–aldosterone system and increasing plasma levels of the vasoconstrictor–angiotensin II at higher doses [2]. This will in return increase the sympathetic activity causing an increase in stroke volume as well as cardiac output due to sympathetic stimulation [3].

Montforte *et al*. [4] studied 107 patients who struggled with alcoholism versus 61 control subjects to assess the functions of the autonomic and peripheral nervous systems in patients. They found that 26 patients (24.3%) had autonomic neuropathy and 34 (32%) had peripheral neuropathy. They concluded that autonomic and peripheral neuropathies are common among patients with alcoholism, and that alcohol is toxic to both autonomic and peripheral nerves in a dose-dependent manner [4]. This is the mechanism that we believe explains our patient’s symptoms as he was consuming over 240 ounces of beer daily for many years. Another study by Matikainen *et al*. [5] was performed on 28 male patients with alcoholism revealing significant polyneuropathy with neurophysiological findings in 7% of subjects. A delay in parasympathetic parameters could be detected in the alcoholics compared with controls.

The most common neuropathy that leads to autonomic dysfunction is diabetes mellitus. However, alcohol-related peripheral neuropathy (ALN) is another important etiology [6]. Uncontrolled diabetes and alcoholism are both present in our patient. In recent studies, failure of thiamine treatment to reverse ALN, together with new information demonstrating clinical and electrophysiological distinctions between ALN and nutritional deficiency neuropathies, suggests that alcohol itself may significantly predispose and enhance development of neuropathy in the appropriate clinical setting [7]. For example, neuropathy can still develop in alcoholics on who are not thiamine deficient [8]. Moreover, subperineurial oedema is more prominent in thiamine deficient neuropathy, whereas segmental de/remyelination is more frequent in alcoholic neuropathy [9]. Our patient reported mild improvement in neuropathy in the follow-up visits after receiving thiamine treatment, similar to Peters *et al*. [10] randomized controlled trial, and after improvement of HbA1c which leads us towards a diagnosis of ALN on top of his already established diabetic peripheral neuropathy.

In conclusion, alcohol metabolites can alter the endothelium leading to changes to the sympathetic and parasympathetic systems which can rarely persist for a long time despite control of risk factors.

## References

[1] Benarroch EE. The central autonomic network: functional organization, dysfunction, and perspective. Mayo Clin Proc 1993;68:988–1001. 8412366 10.1016/s0025-6196(12)62272-1

[2] Frith J, Newton JL. Autonomic dysfunction in chronic liver disease. Liver Int 2009;29:483–9. 10.1111/j.1478-3231.2009.01985.x. 19323779

[3] Gould L, Reddy CV, Becker W, Oh KC, Kim SG. Electrophysiologic properties of alcohol in man. J Electrocardiol 1978;11:219–26. 690548 10.1016/s0022-0736(78)80120-4

[4] Monforte R, Estruch R, Valls-Solé J, Nicolás J, Villalta J, Urbano-Marquez A. Autonomic and peripheral neuropathies in patients with chronic alcoholism. A dose-related toxic effect of alcohol. Arch Neurol 1995;52:45–51. 7826275 10.1001/archneur.1995.00540250049012

[5] Matikainen E, Juntunen J, Salmi T. Autonomic dysfunction in long-standing alcoholism. Alcohol Alcohol Oxf Oxfs 1986;21:69–73. 3954832

[6] Tavee J, Zhou L. Small fiber neuropathy: a burning problem. Cleve Clin J Med 2009;76:297–305. 10.3949/ccjm.76a.08070. 19414545

[7] Mellion M, Gilchrist JM, De La Monte S. Alcohol-related peripheral neuropathy: nutritional, toxic, or both? Muscle Nerve 2011;43:309–16. 10.1002/mus.21946. 21321947 PMC4551507

[8] Behse F, Buchthal F. Alcoholic neuropathy: clinical, electrophysiological, and biopsy findings. Ann Neurol 1977;2:95–110. 10.1002/ana.410020203.

[9] Koike H, Iijima M, Sugiura M, et al. Alcoholic neuropathy is clinicopathologically distinct from thiamine-deficiency neuropathy. Ann Neurol 2003;54:19–29. 10.1002/ana.10550. 12838517

[10] Peters TJ, Kotowicz J, Nyka W, et al. Treatment of alcoholic polyneuropathy with vitamin b complex: a randomized controlled trial. Alcohol Alcohol 2006;41:636–42. 10.1093/alcalc/agl058. 16926172

